# A reverse Mulholland-type inequality in the whole plane

**DOI:** 10.1186/s13660-018-1669-z

**Published:** 2018-04-10

**Authors:** Jianquan Liao, Bicheng Yang

**Affiliations:** Department of Mathematics, Guangdong University of Education, Guangzhou, P.R. China

**Keywords:** 26D15, 47A07, Mulholland-type inequality, Parameter, Weight coefficient, Equivalent form, Reverse

## Abstract

We present a new reverse Mulholland-type inequality in the whole plane with a best possible constant factor by introducing multiparameters, applying weight coefficients, and using the Hermite–Hadamard inequality. Moreover, we consider equivalent forms and some particular cases.

## Introduction

Assuming that $p > 1,\frac{1}{p} + \frac{1}{q} = 1,a_{m},b_{n} \ge 0,0 < \sum_{m = 1}^{\infty} a_{m}^{p} < \infty$, and $0 < \sum_{n = 1}^{\infty} b_{n}^{q} < \infty$, the Hardy–Hilbert inequality is provided as follows (see [1]):

1 $$ \sum_{n = 1}^{\infty} \sum _{m = 1}^{\infty} \frac{a_{m}b_{n}}{m + n} < \frac{\pi}{\sin (\pi /p)} \Biggl( \sum_{m = 1}^{\infty} a_{m}^{p} \Biggr)^{\frac{1}{p}} \Biggl( \sum_{n = 1}^{\infty} b_{n}^{q} \Biggr)^{\frac{1}{q}}, $$

where $\frac{\pi}{\sin (\pi /p)}$ is the best possible constant factor. By Theorem 343 in [1] (replacing $\frac{a_{m}}{m}$ and $\frac{b_{n}}{n}$ by $a _{m}$ and $b _{n}$, respectively), it yields the following Mulholland inequality with the same best value:

2 $$ \sum_{n = 2}^{\infty} \sum _{m = 2}^{\infty} \frac{a_{m}b_{n}}{\ln mn} < \frac{\pi}{\sin (\pi /p)} \Biggl( \sum_{m = 2}^{\infty} \frac{a_{m}^{p}}{m} \Biggr)^{\frac{1}{p}} \Biggl( \sum_{n = 2}^{\infty} \frac{b_{n}^{q}}{n} \Biggr)^{\frac{1}{q}}. $$

Inequalities (1) and (2) play an important role in analysis and its applications (see [1, 2]).

In 2007, Yang [3] published a Hilbert-type integral inequality in the whole plane. Various extensions of (1)–(2) and Yang’s work have been presented since then [4–12]. Recently, Yang and Chen [13] presented the following extension of (1) in the whole plane:

3 $$\begin{aligned} &\sum_{ \vert n \vert = 1}^{\infty} \sum _{ \vert m \vert = 1}^{\infty} \frac{a_{m}b_{n}}{( \vert m - \xi \vert + \vert n - \eta \vert )^{\lambda}} \\ &\quad < 2B(\lambda_{1},\lambda_{2}) \Biggl[ \sum _{ \vert m \vert = 1}^{\infty} \vert m - \xi \vert ^{p(1 - \lambda_{1}) - 1}a_{m}^{p} \Biggr]^{\frac{1}{p}} \Biggl[ \sum _{ \vert n \vert = 1}^{\infty} \vert n - \eta \vert ^{q(1 - \lambda_{2}) - 1} b_{n}^{q} \Biggr]^{\frac{1}{q}}, \end{aligned}$$

where the constant factor $2B(\lambda_{1},\lambda_{2})\ (0 < \lambda_{1},\lambda_{2} \le 1,\lambda_{1} + \lambda_{2} = \lambda,\xi,\eta \in [0,\frac{1}{2}])$ is the best possible. In addition, Xin et al. [14] also carried out a similar result, and Zhong et al. [15] gave the reverse Mulholland’s inequality in the whole plane.

In this paper, we present a new reverse Mulholland-type inequality in the whole plane with a best possible constant factor, which is similar to the results of [13], via introducing multiparameters, applying weight coefficients, and using the Hermite–Hadamard inequality. Moreover, we consider equivalent forms and some particular cases.

## An example and two lemmas

We further assume that $\lambda_{1},\lambda_{2} > 0,\lambda_{1} + \lambda_{2} = \lambda \le 1,\xi,\eta \in [0,\frac{1}{2}], \alpha,\beta \in [\arccos \frac{1}{3},\frac{\pi}{2}]$, and

4 $$ k_{\gamma} (\lambda_{1}): = \frac{2\pi^{2}\csc^{2}\gamma}{ \lambda^{2}\sin^{2}(\frac{\pi \lambda_{1}}{\lambda} )}\quad (\gamma = \alpha,\beta ). $$

### Remark 1

Since $\alpha,\beta \in [\arccos \frac{1}{3},\frac{\pi}{ 2}],\xi,\eta \in [0,\frac{1}{2}]$, it follows that

$$\biggl(\frac{3}{2} \pm \eta \biggr) (1 \mp \cos \beta ) \ge 1 \quad\mbox{and}\quad \biggl(\frac{3}{2} \pm \xi \biggr) (1 \mp \cos \alpha ) \ge 1. $$

### Example 1

We set $g(u): = \frac{\ln u}{u - 1}\ (u > 0),g(1): = \lim_{u \to 1}g(u) = 1$. Then we have $g(u) > 0,g'(u) < 0,g''(u) > 0\ (u > 0)$. By Tailor’s formula we find

$$g(u) = \frac{\ln [1 + (u - 1)]}{u - 1} = \sum_{k = 0}^{\infty} ( - 1)^{k}\frac{(u - 1)^{k}}{k + 1} = \sum_{k = 0}^{\infty} \frac{( - 1)^{k}k!}{k + 1} \frac{(u - 1)^{k}}{k!}\quad ( - 1 < u - 1 \le 1), $$

and then $g^{(k)}(1) = \frac{( - 1)^{k}k!}{k + 1}\ (k = 0,1,2, \ldots )$. Hence, $g^{(0)}(1) = g(1) = 1,g'(1) = - \frac{1}{2},g''(1) = \frac{2}{3}$. It is evident that $g(u) > 0$. We obtain $g'(u) = \frac{h(u)}{u(u - 1)^{2}},h(u): = u - 1 - u\ln u$. Since

$$h'(u) = - \ln u > 0\quad (0 < u < 1);\qquad h'(u) = - \ln u < 0\quad (u > 1), $$

it follows that $h_{\max} = h(1) = 0$ and $h(u) < 0\ (u \ne 1)$. Then we have $g'(u) < 0\ (u \ne 1)$. Since $g'(1) = - \frac{1}{2} < 0$, it follows that $g'(u) < 0\ (u > 0)$. We find

$$g''(u) = \frac{J(u)}{u^{2}(u - 1)^{3}},\quad J(u): = - (u - 1)^{2} - 2u(u - 1) + 2u^{2}\ln u, $$

$J'(u) = - 4(u - 1) + 4u\ln u$, and

$$ J''(u) = 4\ln u < 0\quad (0 < u < 1);\qquad J''(u) = 4\ln u > 0\quad (u > 1). $$

It follows that $J'_{\min} = J'(1) = 0$, $J'(u) > 0\ (u \ne 1)$, and $J(u)$ is strictly increasing. Since $J(1) = 0$, we have

$$J(u) < 0\quad (0 < u < 1);\qquad J(u) > 0\quad (u > 1), $$

and $g''(u) > 0\ (u \ne 1)$. Since $g''(1) = \frac{2}{3} > 0$, we find $g''(u) > 0\ (u > 0)$.

For $0 < \lambda \le 1,0 < \lambda_{2} < 1$, setting $G(u): = g(u^{\lambda} )u^{\lambda_{2} - 1}\ (u > 0)$, we have $G(u) > 0$,

$$\begin{aligned} &G'(u) = \lambda g'\bigl(u^{\lambda} \bigr)u^{\lambda + \lambda_{2} - 2} + (\lambda_{2} - 1)g\bigl(u^{\lambda} \bigr)u^{\lambda_{2} - 2} < 0,\quad\mbox{and} \\ &G''(u) = \lambda^{2}g'' \bigl(u^{\lambda} \bigr)u^{2\lambda + \lambda_{2} - 3} + \lambda (\lambda + \lambda_{2} - 2)g'\bigl(u^{\lambda} \bigr)u^{\lambda + \lambda_{2} - 3} \\ &\phantom{G''(u) =}{}+ \lambda (\lambda_{2} - 1)g'\bigl(u^{\lambda} \bigr)u^{\lambda + \lambda_{2} - 3} + (\lambda_{2} - 1) (\lambda_{2} - 2)g\bigl(u^{\lambda} \bigr)u^{\lambda_{2} - 3} > 0. \end{aligned}$$

We set $F(x,y): = \frac{\ln (x/y)}{x^{\lambda} - y^{\lambda}}\ (\frac{y}{x})^{\lambda_{2} - 1}(x,y > 0)$. Since $F(x,y) = \frac{1}{x^{\lambda}} G(\frac{y}{x})$, we have

$$F(x,y) > 0,\qquad \frac{\partial}{\partial y}F(x,y) < 0,\qquad \frac{\partial^{2}}{\partial y^{2}}F(x,y) > 0. $$

Hence, for $x,y > 1$, we have

$$\frac{1}{y}F(\ln x,\ln y) > 0,\qquad \frac{\partial}{\partial y}\biggl( \frac{1}{y}F(\ln x,\ln y)\biggr) < 0,\qquad \frac{\partial^{2}}{\partial y^{2}}\biggl( \frac{1}{y}F(\ln x,\ln y)\biggr) > 0. $$

### Lemma 1

*If* $f(u) > 0,f'(u) < 0,f''(u) > 0\ (u > \frac{3}{2})$ *and* $\int_{\frac{3}{2}}^{\infty} f(u) \,du < \infty$, *then we have the following Hermite–Hadamard inequality* (*see* [16]):

$$\int_{k}^{k + 1} f(u) \,du < f(k) < \int_{k - \frac{1}{2}}^{k + \frac{1}{2}} f(u) \,du \quad\bigl(k \in \mathbf{N} \backslash \{ 1\} \bigr), $$

*and then*

5 $$ \int_{2}^{\infty} f(u) \,du < \sum _{k = 2}^{\infty} f(k) < \int_{\frac{3}{2}}^{\infty} f(u) \,du. $$

*For* $\vert x \vert , \vert y \vert \ge \frac{3}{2}$, *define*

$$A_{\xi,\alpha} (x): = \vert x - \xi \vert + (x - \xi )\cos \alpha, $$

$A_{\eta,\beta} (y) = \vert y - \eta \vert + (y - \eta )\cos \beta$, *and*

6 $$ k(x,y): = \frac{\ln (\ln A_{\xi,\alpha} (x)/\ln A_{\eta,\beta} (y))}{\ln^{\lambda} A_{\xi,\alpha} (x) - \ln^{\lambda} A_{\eta,\beta} (y)}. $$

*We define two weight coefficients as follows*:

7 $$\begin{aligned} &\omega (\lambda_{2},m): = \sum_{ \vert n \vert = 2}^{\infty} \frac{k(m,n)}{A_{\eta,\beta} (n)} \cdot \frac{\ln^{\lambda_{1}}A_{\xi,\alpha} (m)}{\ln^{1 - \lambda_{2}}A_{\eta,\beta} (n)}, \quad \vert m \vert \in \mathbf{N} \backslash \{ 1\}, \end{aligned}$$

8 $$\begin{aligned} &\varpi (\lambda_{1},n): = \sum_{ \vert m \vert = 2}^{\infty} \frac{k(m,n)}{A_{\xi,\alpha} (m)} \cdot \frac{\ln^{\lambda_{2}}A_{\eta,\beta} (n)}{\ln^{1 - \lambda_{1}}A_{\xi,\alpha} (m)},\quad \vert n \vert \in \mathbf{N} \backslash \{ 1\}, \end{aligned}$$

*where* $\sum_{ \vert j \vert = 2}^{\infty} \cdots = \sum_{j = - 2}^{ - \infty} \cdots+ \sum_{j = 2}^{\infty} \cdots$ ($j = m,n$).

### Lemma 2

*We have the inequalities*

9 $$ k_{\beta} (\lambda_{1}) \bigl(1 - \theta ( \lambda_{2},m)\bigr) < \omega (\lambda_{2},m) < k_{\beta} (\lambda_{1}), \quad \vert m \vert \in \mathbf{N} \backslash \{ 1\}, $$

*where*

10 $$\begin{aligned} \theta (\lambda_{2},m): = {}&\biggl[\frac{\lambda}{\pi} \sin \biggl( \frac{\pi \lambda_{1}}{\lambda} \biggr)\biggr]^{2} \int_{0}^{\frac{\ln [(2 + \eta )(1 + \cos \beta )]}{\ln A_{\xi,\alpha} (m)}} \frac{\ln u}{u^{\lambda} - 1} u^{\lambda_{2} - 1} \,du \\ ={}& O\biggl(\frac{1}{\ln^{\lambda_{2}/2}A_{\xi,\alpha} (m)}\biggr) \in (0,1). \end{aligned}$$

### Proof

For $\vert m \vert \in \mathbf{N}\backslash \{ 1\}$, let

$$\begin{aligned} &k^{(1)}(m,y): = \frac{\ln \ln A_{\xi} (m) - \ln \ln [(y - \eta )(\cos \beta - 1)]}{\ln^{\lambda} A_{\xi} (m) - \ln^{\lambda} [(y - \eta )(\cos \beta - 1)]},\quad y < - \frac{3}{2}, \\ &k^{(2)}(m,y): = \frac{\ln \ln A_{\xi} (m) - \ln \ln [(y - \eta )(\cos \beta + 1)]}{\ln^{\lambda} A_{\xi} (m) - \ln^{\lambda} [(y - \eta )(\cos \beta + 1)]},\quad y > \frac{3}{2}. \end{aligned}$$

Then the equality

$$k^{(1)}(m, - y) = \frac{\ln \ln A_{\xi} (m) - \ln \ln [(y + \eta )(1 - \cos \beta )]}{\ln^{\lambda} A_{\xi} (m) - \ln^{\lambda} [(y + \eta )(1 - \cos \beta )]},\quad y > \frac{3}{2}, $$

yields

11 $$\begin{aligned} \omega (\lambda_{2},m) ={}& \sum_{n = - 2}^{ - \infty} \frac{k^{(1)}(m,n)\ln^{\lambda_{1}}A_{\xi,\alpha} (m)}{(n - \eta )(\cos \beta - 1)\ln^{1 - \lambda_{2}}[(n - \eta )(\cos \beta - 1)]} \\ &{}+ \sum_{n = 2}^{\infty} \frac{k^{(2)}(m,n)\ln^{\lambda_{1}}A_{\xi,\alpha} (m)}{(n - \eta )(1 + \cos \beta )\ln^{1 - \lambda_{2}}[(n - \eta )(1 + \cos \beta )]} \\ ={}& \frac{\ln^{\lambda_{1}}A_{\xi,\alpha} (m)}{1 - \cos \beta} \sum_{n = 2}^{\infty} \frac{k^{(1)}(m, - n)}{(n + \eta )\ln^{1 - \lambda_{2}}[(n + \eta )(1 - \cos \beta )]} \\ &{} + \frac{\ln^{\lambda_{1}}A_{\xi,\alpha} (m)}{1 + \cos \beta} \sum_{n = 2}^{\infty} \frac{k^{(2)}(m,n)}{(n - \eta )\ln^{1 - \lambda_{2}}[(n - \eta )(1 + \cos \beta )]}. \end{aligned}$$

Since $0 < \lambda \le 1,0 < \lambda_{2} < 1$, by Example 1 we find that, for $y > \frac{3}{2}$,

$$\begin{aligned} &\frac{k^{(i)}(m,( - 1)^{i}y)}{(y - ( - 1)^{i}\eta )\ln^{1 - \lambda_{2}}[(y - ( - 1)^{i}\eta )(1 + ( - 1)^{i}\cos \beta )]} > 0, \\ &\frac{d}{dy}\frac{k^{(i)}(m,( - 1)^{i}y)}{(y - ( - 1)^{i}\eta )\ln^{1 - \lambda_{2}}[(y - ( - 1)^{i}\eta )(1 + ( - 1)^{i}\cos \beta )]} < 0, \\ &\frac{d^{2}}{dy^{2}}\frac{k^{(i)}(m,( - 1)^{i}y)}{(y - ( - 1)^{i}\eta )\ln^{1 - \lambda_{2}}[(y - ( - 1)^{i}\eta )(1 + ( - 1)^{i}\cos \beta )]} > 0\quad(i = 1,2), \end{aligned}$$

from which it follows that

$$\frac{k^{(i)}(m,( - 1)^{i}y)}{(y - ( - 1)^{i}\eta )\ln^{1 - \lambda_{2}}[(y - ( - 1)^{i}\eta )(1 + ( - 1)^{i}\cos \beta )]}\quad (i = 1,2) $$

are strictly decreasing and convex in ($\frac{3}{2},\infty $). Then (5) and (11) yield that

$$\begin{aligned} \omega (\lambda_{2},m) < {}&\frac{\ln^{\lambda_{1}}A_{\xi,\alpha} (m)}{1 - \cos \beta} \int_{\frac{3}{2}}^{\infty} \frac{k^{(1)}(m, - y)}{(y + \eta )\ln^{1 - \lambda_{2}}[(y + \eta )(1 - \cos \beta )]} \,dy \\ &{}+ \frac{\ln^{\lambda_{1}}A_{\xi,\alpha} (m)}{1 + \cos \beta} \int_{\frac{3}{2}}^{\infty} \frac{k^{(2)}(m,y)}{(y - \eta )\ln^{1 - \lambda_{2}}[(y - \eta )(1 + \cos \beta )]} \,dy. \end{aligned}$$

Setting $u = \frac{\ln [(y + \eta )(1 - \cos \beta )]}{\ln A_{\xi,\alpha} (m)}\ (u = \frac{\ln [(y - \eta )(1 + \cos \beta )]}{\ln A_{\xi,\alpha} (m)})$ in the first (second) integral, from Remark 1 we obtain

$$\begin{aligned} \omega (\lambda_{2},m) < {}& \biggl(\frac{1}{1 - \cos \beta} + \frac{1}{1 + \cos \beta} \biggr) \int_{0}^{\infty} \frac{\ln u}{u^{\lambda} - 1} u^{\lambda_{2} - 1} \,du \\ ={}& \frac{2\csc^{2}\beta}{\lambda^{2}} \int_{0}^{\infty} \frac{\ln v}{v - 1} v^{(\lambda_{2}/\lambda ) - 1} \,dv = \frac{2\pi^{2}\csc^{2}\beta}{ \lambda^{2}\sin^{2}(\frac{\pi \lambda_{1}}{\lambda} )} = k_{\beta} (\lambda_{1}), \end{aligned}$$

by simplifications. Similarly, (5) and (11) also yield that

$$\begin{aligned} \omega (\lambda_{2},m) >{}& \frac{\ln^{\lambda_{1}}A_{\xi,\alpha} (m)}{1 - \cos \beta} \int_{2}^{\infty} \frac{k^{(1)}(m, - y)}{(y + \eta )\ln^{1 - \lambda_{2}}[(y + \eta )(1 - \cos \beta )]} \,dy \\ &{}+ \frac{\ln^{\lambda_{1}}A_{\xi,\alpha} (m)}{1 + \cos \beta} \int_{2}^{\infty} \frac{k^{(2)}(m,y)}{(y - \eta )\ln^{1 - \lambda_{2}}[(y - \eta )(1 + \cos \beta )]} \,dy \\ \ge{}& \biggl(\frac{1}{1 - \cos \beta} + \frac{1}{1 + \cos \beta} \biggr) \int_{\frac{\ln [(2 + \eta )(1 + \cos \beta )]}{\ln A_{\xi,\alpha} (m)}}^{\infty} \frac{\ln u}{u^{\lambda} - 1} u^{\lambda_{2} - 1} \,du \\ ={}& k_{\beta} (\lambda_{1}) - 2\csc^{2}\beta \int_{0}^{\frac{\ln [(2 + \eta )(1 + \cos \beta )]}{\ln A_{\xi,\alpha} (m)}} \frac{\ln u}{u^{\lambda} - 1}u^{\lambda_{2} - 1} \,du \\ ={}& k_{\beta} (\lambda_{1}) \bigl(1 - \theta ( \lambda_{2},m)\bigr) > 0, \end{aligned}$$

where $\theta (\lambda_{2},m)( < 1)$ is defined in (10). Since

$$\frac{\ln u}{u^{\lambda} - 1}u^{\lambda_{2}/2} \to 0\quad \bigl(u \to 0^{ +} \bigr);\qquad \frac{\ln u}{u^{\lambda} - 1}u^{\lambda_{2}/2} \to \frac{1}{\lambda}\quad (u \to 1), $$

there exists a positive constant *C* such that $\frac{\ln u}{u^{\lambda} - 1}u^{\lambda_{2}/2} \le C\ (0 < u \le 1)$. Then for $A_{\xi,\alpha} (m) \ge (2 + \eta )(1 + \cos \beta )$, we have

12 $$\begin{aligned} 0& < \theta (\lambda_{2},m) \le C\biggl[\frac{\lambda}{\pi} \sin \biggl(\frac{\pi \lambda_{1}}{\lambda} \biggr)\biggr]^{2} \int_{0}^{\frac{\ln [(2 + \eta )(1 + \cos \beta )]}{\ln A_{\xi,\alpha} (m)}} u^{\frac{\lambda_{2}}{2} - 1} \,du \\ &= \frac{2C}{\lambda_{2}}\biggl[\frac{\lambda}{\pi} \sin \biggl(\frac{\pi \lambda_{1}}{\lambda} \biggr)\biggr]^{2}\biggl\{ \frac{\ln [(2 + \eta )(1 + \cos \beta )]}{\ln A_{\xi,\alpha} (m)}\biggr\} ^{\frac{\lambda}{2}}. \end{aligned}$$

Hence, (9) and (10) are valid. □

Similarly, we have the following:

### Lemma 3

*For* $0 < \lambda \le 1,0 < \lambda_{1} < 1$, *we have the inequalities*

13 $$ k_{\alpha} (\lambda_{1}) \bigl(1 - \tilde{\theta} ( \lambda_{1},n)\bigr) < \varpi (\lambda_{1},n) < k_{\alpha} (\lambda_{1}),\quad \vert n \vert \in \mathbf{N} \backslash \{ 1\}, $$

*where*

14 $$\begin{aligned} \tilde{\theta} (\lambda_{1},n): = {}&\biggl[\frac{\lambda}{\pi} \sin \biggl(\frac{\pi \lambda_{1}}{\lambda} \biggr)\biggr]^{2} \int_{0}^{\frac{\ln [(2 + \xi )(1 + \cos \alpha )]}{\ln A_{\eta,\beta} (n)}} \frac{\ln u}{u^{\lambda} - 1} u^{\lambda_{1} - 1} \,du \\ = {}&O\biggl(\frac{1}{\ln^{\lambda_{1}/2}A_{\eta,\beta} (n)}\biggr) \in (0,1). \end{aligned}$$

### Lemma 4

*If* $(\varsigma,\gamma ) = (\xi,\alpha )$ (*or* $(\eta,\beta )$), $\rho > 0$, *then we have*

15 $$ H_{\rho} (\varsigma,\gamma ): = \sum_{ \vert k \vert = 2}^{\infty} \frac{\ln^{ - 1 - \rho} A_{\varsigma,\gamma} (k)}{A_{\varsigma,\gamma} (k)} = \frac{1}{\rho} \bigl(2\csc^{2}\gamma + o(1) \bigr) \quad \bigl(\rho \to 0^{ +} \bigr). $$

### Proof

By (5) we obtain

$$\begin{aligned} H_{\rho} (\varsigma,\gamma ) ={}& \sum_{k = - 2}^{ - \infty} \frac{\ln^{ - 1 - \rho} [(k - \varsigma )(\cos \gamma - 1)]}{(k - \varsigma )(\cos \gamma - 1)} + \sum_{k = 2}^{\infty} \frac{\ln^{ - 1 - \rho} [(k - \varsigma )(\cos \gamma + 1)]}{(k - \varsigma )(\cos \gamma + 1)} \\ = {}&\sum_{k = 2}^{\infty} \biggl\{ \frac{\ln^{ - 1 - \rho} [(k + \varsigma )(1 - \cos \gamma )]}{(k - \varsigma )(1 - \cos \gamma )} + \frac{\ln^{ - 1 - \rho} [(k - \varsigma )(\cos \gamma + 1)]}{(k - \varsigma )(\cos \gamma + 1)}\biggr\} \\ < {}& \int_{\frac{3}{2}}^{\infty} \biggl\{ \frac{\ln^{ - 1 - \rho} [(y + \varsigma )(1 - \cos \gamma )]}{(y - \varsigma )(1 - \cos \gamma )} + \frac{\ln^{ - 1 - \rho} [(y - \varsigma )(\cos \gamma + 1)]}{(y - \varsigma )(\cos \gamma + 1)}\biggr\} \,dy \\ ={}& \frac{1}{\rho} \biggl\{ \frac{\ln^{ - \rho} [(\frac{3}{2} + \varsigma )(1 - \cos \gamma )]}{1 - \cos \gamma} + \frac{\ln^{ - \rho} [(\frac{3}{2} - \varsigma )(1 + \cos \gamma )]}{1 + \cos \gamma} \biggr\} \\ ={}& \frac{1}{\rho} \biggl(\frac{1}{1 - \cos \gamma} + \frac{1}{1 + \cos \gamma} + o_{1}(1)\biggr) = \frac{1}{\rho} \bigl(2\csc^{2}\gamma + o_{1}(1)\bigr) \quad\bigl(\rho \to 0^{ +} \bigr) \end{aligned}$$

and

$$\begin{aligned} H_{\rho} (\varsigma,\gamma ) ={}& \sum_{k = 2}^{\infty} \biggl\{ \frac{\ln^{ - 1 - \rho} [(k + \varsigma )(1 - \cos \gamma )]}{(k - \varsigma )(1 - \cos \gamma )} + \frac{\ln^{ - 1 - \rho} [(k - \varsigma )(\cos \gamma + 1)]}{(k - \varsigma )(\cos \gamma + 1)}\biggr\} \\ >{}& \int_{2}^{\infty} \biggl\{ \frac{\ln^{ - 1 - \rho} [(y + \varsigma )(1 - \cos \gamma )]}{(y - \varsigma )(1 - \cos \gamma )} + \frac{\ln^{ - 1 - \rho} [(y - \varsigma )(\cos \gamma + 1)]}{(y - \varsigma )(\cos \gamma + 1)}\biggr\} \,dy \\ ={}& \frac{1}{\rho} \biggl\{ \frac{\ln^{ - \rho} [(2 + \varsigma )(1 - \cos \gamma )]}{1 - \cos \gamma} + \frac{\ln^{ - \rho} [(2 - \varsigma )(1 + \cos \gamma )]}{1 + \cos \gamma} \biggr\} \\ ={}& \frac{1}{\rho} \biggl(\frac{1}{1 - \cos \gamma} + \frac{1}{1 + \cos \gamma} + o_{2}(1)\biggr) = \frac{1}{\rho} \bigl(2\csc^{2}\gamma + o_{2}(1)\bigr) \quad\bigl(\rho \to 0^{ +} \bigr). \end{aligned}$$

Therefore, (15) is valid. □

## Main results and a few particular cases

### Theorem 1

*Suppose that* $0 < p < 1,\frac{1}{p} + \frac{1}{q} = 1$,

16 $$ k(\lambda_{1}): = k_{\beta}^{1/p}( \lambda_{1})k_{\alpha}^{1/q}(\lambda_{1}) = \frac{2\pi^{2}\csc^{2/p}\beta \csc^{2/q}\alpha}{[\lambda \sin (\frac{\pi \lambda_{1}}{\lambda} )]^{2}}. $$

*If* $a_{m},b_{n} \ge 0\ ( \vert m \vert , \vert n \vert \in \mathbf{N}\backslash \{ 1\} )$, *satisfy*

$$0 < \sum_{ \vert m \vert = 2}^{\infty} \frac{\ln^{p(1 - \lambda_{1}) - 1}A_{\xi,\alpha} (m)}{(A_{\xi,\alpha} (m))^{1 - p}} a_{m}^{p} < \infty,\qquad 0 < \sum_{ \vert n \vert = 2}^{\infty} \frac{\ln^{q(1 - \lambda_{2}) - 1}A_{\eta,\beta} (n)}{(A_{\eta,\beta} (n))^{1 - q}} b_{n}^{q} < \infty, $$

*then for*

$$\begin{aligned} \theta (\lambda_{2},m) &= \biggl[\frac{\lambda}{\pi} \sin \biggl( \frac{\pi \lambda_{1}}{\lambda} \biggr)\biggr]^{2} \int_{0}^{\frac{\ln [(2 + \eta )(1 + \cos \beta )]}{\ln A_{\xi,\alpha} (m)}} \frac{\ln u}{u^{\lambda} - 1} u^{\lambda_{2} - 1} \,du \\ &= O\biggl(\frac{1}{\ln^{\lambda_{2}/2}A_{\xi,\alpha} (m)}\biggr) \in (0,1), \end{aligned}$$

*we obtain the following equivalent reverse Mulholland*-*type inequalities*:

17 $$\begin{aligned} I: ={}& \sum_{ \vert n \vert = 2}^{\infty} \sum _{ \vert m \vert = 2}^{\infty} \frac{\ln (\ln A_{\xi,\alpha} (m)/\ln A_{\eta,\beta} (n))}{\ln^{\lambda} A_{\xi,\alpha} (m) - \ln^{\lambda} A_{\eta,\beta} (n)} a_{m}b_{n} \\ &\quad> \frac{2\pi^{2}\csc^{2/p}\beta \csc^{2/q}\alpha}{[\lambda \sin (\frac{\pi \lambda_{1}}{\lambda} )]^{2}} \\ &\qquad{}\times \Biggl[ \sum_{ \vert m \vert = 2}^{\infty} \bigl(1 - \theta (\lambda_{2},m)\bigr)\frac{\ln^{p(1 - \lambda_{1}) - 1}A_{\xi,\alpha} (m)}{(A_{\xi,\alpha} (m))^{1 - p}}a_{m}^{p} \Biggr]^{\frac{1}{p}} \\ &\qquad{}\times \Biggl[ \sum_{ \vert n \vert = 2}^{\infty} \frac{\ln^{q(1 - \lambda_{2}) - 1}A_{\eta,\beta} (n)}{(A_{\eta,\beta} (n))^{1 - q}}b_{n}^{q} \Biggr]^{\frac{1}{q}}, \end{aligned}$$

18 $$\begin{aligned} J_{1}: ={}& \Biggl\{ \sum_{ \vert n \vert = 2}^{\infty} \frac{\ln^{p\lambda_{2} - 1}A_{\eta,\beta} (n)}{A_{\eta,\beta} (n)} \Biggl[ \sum_{ \vert m \vert = 2}^{\infty} \frac{\ln (\ln A_{\xi,\alpha} (m)/\ln A_{\eta,\beta} (n))}{\ln^{\lambda} A_{\xi,\alpha} (m) - \ln^{\lambda} A_{\eta,\beta} (n)}a_{m} \Biggr]^{p} \Biggr\} ^{\frac{1}{p}} \\ >{}& \frac{2\pi^{2}\csc^{2/p}\beta \csc^{2/q}\alpha}{[\lambda \sin (\frac{\pi \lambda_{1}}{\lambda} )]^{2}} \Biggl[ \sum_{ \vert m \vert = 2}^{\infty} \bigl(1 - \theta (\lambda_{2},m)\bigr)\frac{\ln^{p(1 - \lambda_{1}) - 1}A_{\xi,\alpha} (m)}{(A_{\xi,\alpha} (m))^{1 - p}}a_{m}^{p} \Biggr]^{\frac{1}{p}}, \end{aligned}$$

19 $$\begin{aligned} J_{2}: = {}&\Biggl[ \sum_{ \vert m \vert = 2}^{\infty} \frac{\ln^{q\lambda_{1} - 1}A_{\xi,\alpha} (m)}{(1 - \theta (\lambda_{2},m))^{q - 1}A_{\xi,\alpha} (m)} \Biggl( \sum_{ \vert n \vert = 2}^{\infty} \frac{\ln (\ln A_{\xi,\alpha} (m)/\ln A_{\eta,\beta} (n))}{\ln^{\lambda} A_{\xi,\alpha} (m) - \ln^{\lambda} A_{\eta,\beta} (n)}b_{n} \Biggr)^{q} \Biggr]^{\frac{1}{q}} \\ >{}& \frac{2\pi^{2}\csc^{2/p}\beta \csc^{2/q}\alpha}{[\lambda \sin (\frac{\pi \lambda_{1}}{\lambda} )]^{2}} \Biggl[ \sum_{ \vert n \vert = 2}^{\infty} \frac{\ln^{q(1 - \lambda_{2}) - 1}A_{\eta,\beta} (n)}{(A_{\eta,\beta} (n))^{1 - q}}b_{n}^{q} \Biggr]^{\frac{1}{q}}. \end{aligned}$$

*Particularly*, (i) *for* $\alpha = \beta = \frac{\pi}{2},\xi,\eta \in [0,\frac{1}{2}]$, *setting*

$$\begin{aligned} \theta_{1}(\lambda_{2},m)&: = \biggl[\frac{\lambda}{\pi} \sin \biggl(\frac{\pi \lambda_{1}}{\lambda} \biggr)\biggr]^{2} \int_{0}^{\frac{\ln (2 + \eta )}{\ln \vert m - \xi \vert }} \frac{\ln u}{u^{\lambda} - 1} u^{\lambda_{2} - 1} \,du \\ &= O\biggl(\frac{1}{\ln^{\lambda_{2}/2} \vert m - \xi \vert }\biggr) \in (0,1), \end{aligned}$$

*we have the following equivalent reverse Mulholland*-*type inequalities*:

20 $$\begin{aligned} &\sum_{ \vert n \vert = 2}^{\infty} \sum _{ \vert m \vert = 2}^{\infty} \frac{\ln ( \vert m - \xi \vert / \vert n - \eta \vert )}{\ln^{\lambda} \vert m - \xi \vert - \ln^{\lambda} \vert n - \eta \vert } a_{m}b_{n} \\ &\quad > \frac{2\pi^{2}}{[\lambda \sin (\frac{\pi \lambda_{1}}{\lambda} )]^{2}} \\ &\qquad{}\times \Biggl[ \sum_{ \vert m \vert = 2}^{\infty} \bigl(1 - \theta_{1}(\lambda_{2},m)\bigr)\frac{\ln^{p(1 - \lambda_{1}) - 1} \vert m - \xi \vert }{ \vert m - \xi \vert ^{1 - p}}a_{m}^{p} \Biggr]^{\frac{1}{p}} \\ &\qquad{}\times\Biggl[ \sum_{ \vert n \vert = 2}^{\infty} \frac{\ln^{q(1 - \lambda_{2}) - 1} \vert n - \eta \vert }{ \vert n - \eta \vert ^{1 - q}}b_{n}^{q} \Biggr]^{\frac{1}{q}}, \end{aligned}$$

21 $$\begin{aligned} &\Biggl[ \sum_{ \vert n \vert = 2}^{\infty} \frac{\ln^{p\lambda_{2} - 1} \vert n - \eta \vert }{ \vert n - \eta \vert } \Biggl( \sum_{ \vert m \vert = 2}^{\infty} \frac{\ln ( \vert m - \xi \vert / \vert n - \eta \vert )}{\ln^{\lambda} \vert m - \xi \vert - \ln^{\lambda} \vert n - \eta \vert }a_{m} \Biggr)^{p} \Biggr]^{\frac{1}{p}} \\ &\quad > \frac{2\pi^{2}}{[\lambda \sin (\frac{\pi \lambda_{1}}{\lambda} )]^{2}} \Biggl[ \sum_{ \vert m \vert = 2}^{\infty} \bigl(1 - \theta_{1}(\lambda_{2},m)\bigr) \frac{\ln^{p(1 - \lambda_{1}) - 1} \vert m - \xi \vert }{ \vert m - \xi \vert ^{1 - p}}a_{m}^{p} \Biggr]^{\frac{1}{p}}, \end{aligned}$$

22 $$\begin{aligned} &\Biggl[ \sum_{ \vert m \vert = 2}^{\infty} \frac{\ln^{q\lambda_{1} - 1} \vert m - \xi \vert }{(1 - \theta_{1}(\lambda_{2},m))^{q - 1} \vert m - \xi \vert } \Biggl( \sum_{ \vert n \vert = 2}^{\infty} \frac{\ln ( \vert m - \xi \vert / \vert n - \eta \vert )a_{m}}{\ln^{\lambda} \vert m - \xi \vert - \ln^{\lambda} \vert n - \eta \vert }b_{n} \Biggr)^{q} \Biggr]^{\frac{1}{q}} \\ &\quad > \frac{2\pi^{2}}{[\lambda \sin (\frac{\pi \lambda_{1}}{\lambda} )]^{2}} \Biggl[ \sum_{ \vert n \vert = 2}^{\infty} \frac{\ln^{q(1 - \lambda_{2}) - 1} \vert n - \eta \vert }{ \vert n - \eta \vert ^{1 - q}}b_{n}^{q} \Biggr]^{\frac{1}{q}}. \end{aligned}$$

(ii) *For* $\xi = \eta = 0,\alpha,\beta \in [\arccos \frac{1}{3},\frac{\pi}{ 2}]$, *setting*

$$\begin{aligned} \theta_{2}(\lambda_{2},m): ={}& \biggl[\frac{\lambda}{\pi} \sin \biggl(\frac{\pi \lambda_{1}}{\lambda} \biggr)\biggr]^{2} \int_{0}^{\frac{\ln 2(1 + \cos \beta )}{\ln ( \vert m \vert + m\cos \alpha )}} \frac{\ln u}{u^{\lambda} - 1} u^{\lambda_{2} - 1} \,du \\ ={}& O\biggl(\frac{1}{\ln^{\lambda_{2}/2}A_{\xi,\alpha} (m)}\biggr) \in (0,1), \end{aligned}$$

*we have the following equivalent reverse Mulholland*-*type inequalities*:

23 $$\begin{aligned} &\sum_{ \vert n \vert = 2}^{\infty} \sum _{ \vert m \vert = 2}^{\infty} \frac{\ln [\ln ( \vert m \vert + m\cos \alpha )/\ln ( \vert n \vert + n\cos \beta )]}{\ln^{\lambda} ( \vert m \vert + m\cos \alpha ) - \ln^{\lambda} ( \vert n \vert + n\cos \beta )} a_{m}b_{n} \\ &\quad > \frac{2\pi^{2}\csc^{2/p}\beta \csc^{2/q}\alpha}{[\lambda \sin (\frac{\pi \lambda_{1}}{\lambda} )]^{2}} \\ &\qquad{}\times \Biggl[ \sum_{ \vert m \vert = 2}^{\infty} \bigl(1 - \theta_{2}(\lambda_{2},m)\bigr)\frac{\ln^{p(1 - \lambda_{1}) - 1}( \vert m \vert + m\cos \alpha )}{( \vert m \vert + m\cos \alpha )^{1 - p}}a_{m}^{p} \Biggr]^{\frac{1}{p}} \\ &\qquad{}\times\Biggl[ \sum_{ \vert n \vert = 2}^{\infty} \frac{\ln^{q(1 - \lambda_{2}) - 1}( \vert n \vert + n\cos \beta )}{( \vert n \vert + n\cos \beta )^{1 - q}}b_{n}^{q} \Biggr]^{\frac{1}{q}}, \end{aligned}$$

24 $$\begin{aligned} &\Biggl\{ \sum_{ \vert n \vert = 2}^{\infty} \frac{\ln^{p\lambda_{2} - 1}( \vert n \vert + n\cos \beta )}{ \vert n \vert + n\cos \beta} \Biggl[ \sum_{ \vert m \vert = 2}^{\infty} \frac{\ln [\ln ( \vert m \vert + m\cos \alpha )/\ln ( \vert n \vert + n\cos \beta )]}{\ln^{\lambda} ( \vert m \vert + m\cos \alpha ) - \ln^{\lambda} ( \vert n \vert + n\cos \beta )}a_{m} \Biggr]^{p} \Biggr\} ^{\frac{1}{p}} \\ &\quad> \frac{2\pi^{2}\csc^{2/p}\beta \csc^{2/q}\alpha}{[\lambda \sin (\frac{\pi \lambda_{1}}{\lambda} )]^{2}} \Biggl[ \sum_{ \vert m \vert = 2}^{\infty} \bigl(1 - \theta_{2}(\lambda_{2},m)\bigr) \frac{\ln^{p(1 - \lambda_{1}) - 1}( \vert m \vert + m\cos \alpha )}{( \vert m \vert + m\cos \alpha )^{1 - p}}a_{m}^{p} \Biggr]^{\frac{1}{p}}, \end{aligned}$$

25 $$\begin{aligned} &\Biggl[ \sum_{ \vert m \vert = 2}^{\infty} \frac{\ln^{q\lambda_{1} - 1}( \vert m \vert + m\cos \alpha )}{(1 - \theta_{2}(\lambda_{2},m))^{q - 1}( \vert m \vert + m\cos \alpha )} \\ &\qquad{}\times \Biggl( \sum_{ \vert n \vert = 2}^{\infty} \frac{\ln [\ln ( \vert m \vert + m\cos \alpha )/\ln ( \vert n \vert + n\cos \beta )]}{\ln^{\lambda} ( \vert m \vert + m\cos \alpha ) - \ln^{\lambda} ( \vert n \vert + n\cos \beta )}b_{n} \Biggr)^{q} \Biggr]^{\frac{1}{q}} \\ &\quad > \frac{2\pi^{2}\csc^{2/p}\beta \csc^{2/q}\alpha}{[\lambda \sin (\frac{\pi \lambda_{1}}{\lambda} )]^{2}} \Biggl[ \sum_{ \vert n \vert = 2}^{\infty} \frac{\ln^{q(1 - \lambda_{2}) - 1}( \vert n \vert + n\cos \beta )}{( \vert n \vert + n\cos \beta )^{1 - q}}b_{n}^{q} \Biggr]^{\frac{1}{q}}. \end{aligned}$$

### Proof

Applying the reverse Hölder inequality with weight (see [17]) and (8), we find

$$\begin{aligned} &\Biggl( \sum_{ \vert m \vert = 2}^{\infty} k(m,n)a_{m} \Biggr)^{p} \\ &\quad= \Biggl\{ \sum_{ \vert m \vert = 2}^{\infty} k(m,n) \biggl[ \frac{(A_{\xi,\alpha} (m))^{\frac{1}{q}}\ln^{\frac{1 - \lambda_{1}}{q}}A_{\xi,\alpha} (m)}{\ln^{\frac{1 - \lambda_{2}}{p}}A_{\eta,\beta} (n)}a_{m} \biggr] \biggl[ \frac{\ln^{\frac{1 - \lambda_{2}}{p}}A_{\eta,\beta} (n)}{(A_{\xi,\alpha} (m))^{\frac{1}{q}}\ln^{\frac{1 - \lambda_{1}}{q}}A_{\xi,\alpha} (m)} \biggr] \Biggr\} ^{p} \\ &\quad\ge \sum_{ \vert m \vert = 2}^{\infty} k(m,n) \frac{(A_{\xi,\alpha} (m))^{\frac{p}{q}}\ln^{\frac{(1 - \lambda_{1})p}{q}}A_{\xi,\alpha} (m)}{\ln^{1 - \lambda_{2}}A_{\eta,\beta} (n)}a_{m}^{p} \\ &\qquad{}\times\Biggl[ \sum _{ \vert m \vert = 2}^{\infty} k(m,n)\frac{\ln^{\frac{(1 - \lambda_{2})q}{p}}A_{\eta,\beta} (n)}{A_{\xi,\alpha} (m)\ln^{1 - \lambda_{1}}A_{\xi,\alpha} (m)} \Biggr]^{p - 1} \\ &\quad= \frac{(\varpi (\lambda_{1},n))^{p - 1}A_{\eta,\beta} (n)}{\ln^{p\lambda_{2} - 1}A_{\eta,\beta} (n)}\sum_{ \vert m \vert = 2}^{\infty} k(m,n) \frac{(A_{\xi,\alpha} (m))^{\frac{p}{q}}\ln^{\frac{(1 - \lambda_{1})p}{q}}A_{\xi,\alpha} (m)}{A_{\eta,\beta} (n)\ln^{1 - \lambda_{2}}A_{\eta,\beta} (n)}a_{m}^{p}. \end{aligned}$$

Then since $0 < p < 1$, by (13) this yields

26 $$\begin{aligned} J &> k_{\alpha}^{1/q}(\lambda_{1}) \Biggl[ \sum _{ \vert n \vert = 2}^{\infty} \sum _{ \vert m \vert = 2}^{\infty} k(m,n)\frac{(A_{\xi,\alpha} (m))^{\frac{p}{q}}\ln^{\frac{(1 - \lambda_{1})p}{q}}A_{\xi,\alpha} (m)}{A_{\eta,\beta} (n)\ln^{1 - \lambda_{2}}A_{\eta,\beta} (n)}a_{m}^{p} \Biggr]^{\frac{1}{p}} \\ &= k_{\alpha}^{1/q}(\lambda_{1}) \Biggl[ \sum _{ \vert m \vert = 2}^{\infty} \sum _{ \vert n \vert = 2}^{\infty} k(m,n)\frac{(A_{\xi,\alpha} (m))^{\frac{p}{q}}\ln^{\frac{(1 - \lambda_{1})p}{q}}A_{\xi,\alpha} (m)}{A_{\eta,\beta} (n)\ln^{1 - \lambda_{2}}A_{\eta,\beta} (n)}a_{m}^{p} \Biggr]^{\frac{1}{p}} \\ &= k_{\alpha}^{1/q}(\lambda_{1}) \Biggl[ \sum _{ \vert m \vert = 2}^{\infty} \omega (\lambda_{2},m) \frac{n^{p(1 - \lambda_{1}) - 1}A_{\xi,\alpha} (m)}{(A_{\xi,\alpha} (m))^{1 - p}}a_{m}^{p} \Biggr]^{\frac{1}{p}}. \end{aligned}$$

Combining (9) and (16), we obtain (18).

Using the reverse Hölders inequality again, we obtain

27 $$\begin{aligned} I &= \sum_{ \vert n \vert = 2}^{\infty} \Biggl[ \frac{(A_{\eta,\beta} (n))^{\frac{ - 1}{p}}}{\ln^{\frac{1}{p} - \lambda_{2}}A_{\eta,\beta} (n)}\sum_{ \vert m \vert = 2}^{\infty} k(m,n)a_{m} \Biggr] \biggl[ \frac{\ln^{\frac{1}{p} - \lambda_{2}}A_{\eta,\beta} (n)}{(A_{\eta,\beta} (n))^{\frac{ - 1}{p}}}b_{n} \biggr] \\ &\ge J_{1} \Biggl[ \sum_{ \vert n \vert = 2}^{\infty} \frac{\ln^{q(1 - \lambda_{2}) - 1}A_{\eta,\beta} (n)}{(A_{\eta,\beta} (n))^{1 - q}} b_{n}^{q} \Biggr]^{\frac{1}{q}}. \end{aligned}$$

Then by (18) we obtain (17).

On the other-hand, assuming that (17) is valid, letting

$$b_{n}: = \frac{\ln^{p\lambda_{2} - 1}A_{\eta,\beta} (n)}{A_{\eta,\beta} (n)} \Biggl( \sum _{ \vert m \vert = 2}^{\infty} k(m,n)a_{m} \Biggr)^{p - 1}, \quad \vert n \vert \in \mathbf{N}\backslash \{ 1\}, $$

we find

$$J_{1} = \Biggl[ \sum_{ \vert n \vert = 2}^{\infty} \frac{\ln^{q(1 - \lambda_{2}) - 1}A_{\eta,\beta} (n)}{(A_{\eta,\beta} (n))^{1 - q}} b_{n}^{q} \Biggr]^{\frac{1}{p}}. $$

By (26) it follows that $J_{1} > 0$. If $J_{1} = \infty$, then (19) is trivially valid; if $J_{1} < \infty$, then by (17) we have

$$\begin{aligned} &\sum_{ \vert n \vert = 2}^{\infty} \frac{\ln^{q(1 - \lambda_{2}) - 1}A_{\eta,\beta} (n)}{(A_{\eta,\beta} (n))^{1 - q}} b_{n}^{q}\\ &\quad = J_{1}^{p} = I \\ &\quad> k(\lambda_{1}) \Biggl[ \sum_{ \vert m \vert = 2}^{\infty} \bigl(1 - \theta (\lambda_{2},m)\bigr)\frac{\ln^{p(1 - \lambda_{1}) - 1}A_{\xi,\alpha} (m)}{(A_{\xi,\alpha} (m))^{1 - p}}a_{m}^{p} \Biggr]^{\frac{1}{p}} \Biggl[ \sum_{ \vert n \vert = 2}^{\infty} \frac{\ln^{q(1 - \lambda_{2}) - 1}A_{\eta,\beta} (n)}{(A_{\eta,\beta} (n))^{1 - q}}b_{n}^{q} \Biggr]^{\frac{1}{q}}, \\ &J_{1} = \Biggl[ \sum_{ \vert n \vert = 2}^{\infty} \frac{\ln^{q(1 - \lambda_{2}) - 1}A_{\eta,\beta} (n)}{(A_{\eta,\beta} (n))^{1 - q}} b_{n}^{q} \Biggr]^{\frac{1}{p}} > k( \lambda_{1}) \Biggl[ \sum_{ \vert m \vert = 2}^{\infty} \bigl(1 - \theta (\lambda_{2},m)\bigr)\frac{\ln^{p(1 - \lambda_{1}) - 1}A_{\xi,\alpha} (m)}{(A_{\xi,\alpha} (m))^{1 - p}}a_{m}^{p} \Biggr]^{\frac{1}{p}}. \end{aligned}$$

Thus (18) is valid, which is equivalent to (17).

We further prove that (19) is equivalent to (17). Using the reverse Hölders inequality, we have

28 $$\begin{aligned} I={}& \sum_{ \vert m \vert = 2}^{\infty} \biggl[ \bigl(1 - \theta (\lambda_{2},m)\bigr)^{\frac{1}{p}}\frac{\ln^{\frac{1}{q} - \lambda_{1}}A_{\xi,\alpha} (m)}{(A_{\xi,\alpha} (m))^{\frac{ - 1}{q}}}a_{m} \biggr] \\ &{}\times \Biggl[ \frac{n^{\frac{ - 1}{q} + \lambda_{1}}A_{\xi,\alpha} (m)}{(1 - \theta (\lambda_{2},m))^{\frac{1}{p}}(A_{\xi,\alpha} (m))^{\frac{1}{q}}}\sum_{ \vert n \vert = 2}^{\infty} k(m,n)b_{n} \Biggr] \\ \ge{}& \Biggl[ \sum_{ \vert m \vert = 2}^{\infty} \bigl(1 - \theta (\lambda_{2},m)\bigr)\frac{\ln^{p(1 - \lambda_{1}) - 1}A_{\xi,\alpha} (m)}{(A_{\xi,\alpha} (m))^{1 - p}}a_{m}^{p} \Biggr]^{\frac{1}{p}}J_{2}, \end{aligned}$$

and then (19) is valid by (17).

On the other-hand, assuming that (17) is valid, we set

$$a_{m}: = \frac{\ln^{q\lambda_{1} - 1}A_{\xi,\alpha} (m)}{(1 - \theta (\lambda_{2},m))^{q - 1}A_{\xi,\alpha} (m)} \Biggl( \sum _{ \vert n \vert = 2}^{\infty} \frac{\ln (\ln A_{\xi,\alpha} (m)/\ln A_{\eta,\beta} (n))}{\ln^{\lambda} A_{\xi,\alpha} (m) - \ln^{\lambda} A_{\eta,\beta} (n)}b_{n} \Biggr)^{q - 1},\quad m \in \mathbf{N}\backslash \{ 1\}, $$

and find

$$J_{2} = \Biggl[ \sum_{ \vert m \vert = 2}^{\infty} \bigl(1 - \theta (\lambda_{2},m)\bigr)\frac{\ln^{p(1 - \lambda_{1}) - 1}A_{\xi,\alpha} (m)}{(A_{\xi,\alpha} (m))^{1 - p}}a_{m}^{p} \Biggr]^{\frac{1}{q}}. $$

If $J_{2} = 0$, then (19) is impossible, so that $J_{2} > 0$. If $J_{2} = \infty$, then (19) is trivially valid; if $J_{2} < \infty$, then by (17) we have

$$\begin{aligned} &\sum_{ \vert m \vert = 2}^{\infty} \bigl(1 - \theta ( \lambda_{2},m)\bigr)\frac{\ln^{p(1 - \lambda_{1}) - 1}A_{\xi,\alpha} (m)}{(A_{\xi,\alpha} (m))^{1 - p}}a_{m}^{p} \\ &\quad= J_{2}^{q} = I \\ &\quad> k(\lambda_{1}) \Biggl[ \sum_{ \vert m \vert = 2}^{\infty} \bigl(1 - \theta (\lambda_{2},m)\bigr)\frac{\ln^{p(1 - \lambda_{1}) - 1}A_{\xi,\alpha} (m)}{(A_{\xi,\alpha} (m))^{1 - p}}a_{m}^{p} \Biggr]^{\frac{1}{p}} \Biggl[ \sum_{ \vert n \vert = 2}^{\infty} \frac{\ln^{q(1 - \lambda_{2}) - 1}A_{\eta,\beta} (n)}{(A_{\eta,\beta} (n))^{1 - q}}b_{n}^{q} \Biggr]^{\frac{1}{q}}, \\ &\Biggl[ \sum_{ \vert m \vert = 2}^{\infty} \bigl(1 - \theta ( \lambda_{2},m)\bigr)\frac{\ln^{p(1 - \lambda_{1}) - 1}A_{\xi,\alpha} (m)}{(A_{\xi,\alpha} (m))^{1 - p}}a_{m}^{p} \Biggr]^{\frac{1}{q}} \\ &\quad= J_{2} \\ &\quad > k(\lambda_{1}) \Biggl[ \sum_{ \vert n \vert = 2}^{\infty} \frac{\ln^{q(1 - \lambda_{2}) - 1}A_{\eta,\beta} (n)}{(A_{\eta,\beta} (n))^{1 - q}}b_{n}^{q} \Biggr]^{\frac{1}{q}}. \end{aligned}$$

Thus (19) is valid, which is equivalent to (17).

Hence, inequalities (17), (18), and (19) are equivalent. □

### Theorem 2

*Under the assumptions in Theorem *1,

$$k(\lambda_{1}) = \frac{2\pi^{2}\csc^{2/p}\beta \csc^{2/q}\alpha}{[\lambda \sin (\frac{\pi \lambda_{1}}{\lambda} )]^{2}} $$

*is the best possible constant factor in* (17), (18), *and* (19).

### Proof

For $0 < \varepsilon < \min \{ p\lambda_{1},p(1 - \lambda_{2})\}$, we set $\tilde{\lambda}_{1} = \lambda_{1} - \frac{\varepsilon}{p}( \in (0,1)),\tilde{\lambda}_{2} = \lambda_{2} + \frac{\varepsilon}{p} ( \in (0,1))$, and

$$\begin{aligned} &\tilde{a}_{m}: = \frac{\ln^{\lambda_{1} - \frac{\varepsilon}{p} - 1}A_{\xi,\alpha} (m)}{A_{\xi,\alpha} (m)} = \frac{\ln^{\tilde{\lambda}_{1} - 1}A_{\xi,\alpha} (m)}{A_{\xi,\alpha} (m)}\quad \bigl( \vert m \vert \in \mathbf{N}\backslash \{ 1\} \bigr), \\ &\tilde{b}_{n}: = \frac{\ln^{\lambda_{2} - \frac{\varepsilon}{q} - 1}A_{\eta,\beta} (n)}{A_{\eta,\beta} (n)} = \frac{\ln^{\tilde{\lambda}_{2} - \varepsilon - 1}A_{\eta,\beta} (n)}{A_{\eta,\beta} (n)}\quad \bigl( \vert n \vert \in \mathbf{N}\backslash \{ 1\} \bigr). \end{aligned}$$

By (15) and (13) we find

$$\begin{aligned} &\tilde{I}_{2}: = \Biggl[ \sum_{ \vert m \vert = 2}^{\infty} \bigl(1 - \theta (\lambda_{2},m)\bigr)\frac{\ln^{p(1 - \lambda_{1}) - 1}A_{\xi,\alpha} (m)}{(A_{\xi,\alpha} (m))^{1 - p}} \tilde{a}_{m}^{p} \Biggr]^{\frac{1}{p}} \Biggl[ \sum _{ \vert n \vert = 2}^{\infty} \frac{\ln^{q(1 - \lambda_{2}) - 1}A_{\eta,\beta} (n)}{(A_{\eta,\beta} (n))^{1 - q}} \tilde{b}_{n}^{q} \Biggr]^{\frac{1}{q}} \\ &\phantom{\tilde{I}_{2}:}= \Biggl[ \sum_{ \vert m \vert = 2}^{\infty} \frac{\ln^{ - 1 - \varepsilon} A_{\xi,\alpha} (m)}{A_{\xi,\alpha} (m)} - \sum_{ \vert m \vert = 2}^{\infty} \frac{O(\ln^{ - 1 - (\frac{\lambda_{2}}{2} + \varepsilon )}A_{\xi,\alpha} (m))}{A_{\xi,\alpha} (m)} \Biggr]^{\frac{1}{p}} \Biggl[ \sum _{ \vert n \vert = 2}^{\infty} \frac{\ln^{ - 1 - \varepsilon} A_{\eta,\beta} (n)}{A_{\eta,\beta} (n)} \Biggr]^{\frac{1}{q}} \\ &\phantom{\tilde{I}_{2}:}= \frac{1}{\varepsilon} \bigl(2\csc^{2}\alpha + o(1) - \varepsilon O(1) \bigr)^{\frac{1}{p}}\bigl(2\csc^{2}\beta + \tilde{o}(1) \bigr)^{\frac{1}{q}}\quad\bigl(\varepsilon \to 0^{ +} \bigr), \\ &\tilde{I} = \sum_{ \vert n \vert = 2}^{\infty} \sum _{ \vert m \vert = 2}^{\infty} k(m,n) \tilde{a}_{m} \tilde{b}_{n} = \sum_{ \vert m \vert = 2}^{\infty} \sum_{ \vert n \vert = 2}^{\infty} k(m,n) \frac{\ln^{\tilde{\lambda}_{1} - 1}A_{\xi,\alpha} (m)}{A_{\xi,\alpha} (m)} \frac{\ln^{\tilde{\lambda}_{2} - \varepsilon - 1}A_{\eta,\beta} (n)}{A_{\eta,\beta} (n)} \\ &\phantom{\tilde{I} =}= \sum_{ \vert n \vert = 2}^{\infty} \varpi (\tilde{ \lambda}_{1},n)\frac{\ln^{ - 1 - \varepsilon} A_{\eta,\beta} (n)}{A_{\eta,\beta} (n)} < k_{\alpha} (\tilde{ \lambda}_{1})\sum_{ \vert n \vert = 2}^{\infty} \frac{\ln^{ - 1 - \varepsilon} A_{\eta,\beta} (n)}{A_{\eta,\beta} (n)} \\ &\phantom{\tilde{I} =}= \frac{1}{\varepsilon} k_{\alpha} (\tilde{\lambda}_{1}) \bigl(2\csc^{2}\beta + o(1)\bigr). \end{aligned}$$

If there exists a positive number $k \ge k(\lambda_{1})$ such that (17) is still valid when replacing $k(\lambda_{1})$ by *k*, then, in particular, we have

$$\varepsilon \tilde{I} = \varepsilon \sum_{ \vert m \vert = 2}^{\infty} \sum_{ \vert n \vert = 2}^{\infty} k(m,n) \tilde{a}_{m} \tilde{b}_{n} > \varepsilon k\tilde{I}_{2}. $$

We obtain from the previous results that

$$\begin{aligned} &k_{\beta} \biggl(\lambda_{1} + \frac{\varepsilon}{ q}\biggr) \bigl(2\csc^{2}\alpha + o(1)\bigr) \\ &\quad > k\bigl(2\csc^{2}\alpha + o(1) - \varepsilon O(1) \bigr)^{\frac{1}{p}}\bigl(2\csc^{2}\beta + \tilde{o}(1) \bigr)^{\frac{1}{q}}, \end{aligned}$$

and then

$$\frac{4\pi^{2}}{\lambda^{2}\sin^{2}(\frac{\pi \lambda_{1}}{\lambda} )}\csc^{2}\beta \csc^{2}\alpha \ge 2k \csc^{\frac{2}{p}}\alpha \csc^{\frac{2}{q}}\beta \quad \bigl(\varepsilon \to 0^{ +} \bigr), $$

namely, $k(\lambda_{1}) = \frac{2\pi^{2}}{\lambda^{2}\sin^{2}(\frac{\pi \lambda_{1}}{\lambda} )}\csc^{\frac{2}{p}}\beta \csc^{\frac{2}{q}}\alpha \ge k$. Hence, $k = k(\lambda_{1})$ is the best possible constant factor of (17).

The constant factor $k(\lambda_{1})$ in (18) and (19) is still the best possible. Otherwise, we would reach a contradiction by (27) and (28) that the constant factor in (17) is not the best possible. □

### Remark 2

(i) For $\xi = \eta = 0$ in (20), setting

$$\tilde{\theta}_{1}(\lambda_{2},m): = \biggl[ \frac{\lambda}{\pi} \sin \biggl(\frac{\pi \lambda_{1}}{\lambda} \biggr)\biggr]^{2} \int_{0}^{\frac{\ln 2}{\ln \vert m \vert }} \frac{\ln u}{u^{\lambda} - 1} u^{\lambda_{2} - 1} \,du = O\biggl(\frac{1}{\ln^{\lambda_{2}/2} \vert m \vert }\biggr) \in (0,1), $$

we have the following new inequality:

29 $$\begin{aligned} &\sum_{ \vert n \vert = 2}^{\infty} \sum _{ \vert m \vert = 2}^{\infty} \frac{\ln (\ln \vert m \vert /\ln \vert n \vert )}{\ln^{\lambda} \vert m \vert - \ln^{\lambda} \vert n \vert } a_{m}b_{n} \\ &\quad > \frac{2\pi^{2}}{[\lambda \sin (\frac{\pi \lambda_{1}}{\lambda} )]^{2}} \\ &\qquad{}\times \Biggl[ \sum_{ \vert m \vert = 2}^{\infty} \bigl(1 - \tilde{\theta}_{1}(\lambda_{2},m)\bigr) \frac{\ln^{p(1 - \lambda_{1}) - 1} \vert m \vert }{ \vert m \vert ^{1 - p}}a_{m}^{p} \Biggr]^{\frac{1}{p}} \Biggl[ \sum_{ \vert n \vert = 2}^{\infty} \frac{\ln^{q(1 - \lambda_{2}) - 1} \vert n \vert }{ \vert n \vert ^{1 - q}}b_{n}^{q} \Biggr]^{\frac{1}{q}}. \end{aligned}$$

It follows that (20) is an extension of (29). In particular, for $\lambda = 1,\lambda_{1} = \lambda_{2} = \frac{1}{2}$, setting

$$\tilde{\theta}_{1}(m): = \frac{1}{\pi^{2}} \int_{0}^{\frac{\ln 2}{\ln \vert m \vert }} \frac{\ln u}{u - 1} u^{\frac{ - 1}{2}} \,du = O\biggl(\frac{1}{\ln^{1/4} \vert m \vert }\biggr) \in (0,1), $$

we have the following simple reverse Mulholland-type inequality in the whole plane:

30 $$\begin{aligned} &\sum_{ \vert n \vert = 2}^{\infty} \sum _{ \vert m \vert = 2}^{\infty} \frac{\ln (\ln \vert m \vert /\ln \vert n \vert )}{\ln ( \vert m \vert / \vert n \vert )} a_{m}b_{n} \\ &\quad> 2\pi^{2} \Biggl[ \sum_{ \vert m \vert = 2}^{\infty} \bigl(1 - \tilde{\theta}_{1}(m)\bigr)\frac{\ln^{\frac{p}{2} - 1} \vert m \vert }{ \vert m \vert ^{1 - p}}a_{m}^{p} \Biggr]^{\frac{1}{p}} \Biggl( \sum_{ \vert n \vert = 2}^{\infty} \frac{\ln^{\frac{q}{2} - 1} \vert n \vert }{ \vert n \vert ^{1 - q}}b_{n}^{q} \Biggr)^{\frac{1}{q}}. \end{aligned}$$

(ii) If $a_{ - m} = a_{m},b_{ - n} = b_{n}\ (m,n \in \mathbf{N}\backslash \{ 1\} )$, for $m \in \mathbf{N}\backslash \{ 1\}$, setting

$$\begin{aligned} &\stackrel{\frown}{\theta}_{1}(\lambda_{2},m): = \biggl[ \frac{\lambda}{\pi} \sin \biggl(\frac{\pi \lambda_{1}}{\lambda} \biggr)\biggr]^{2} \int_{0}^{\frac{\ln (2 + \eta )}{\ln (m - \xi )}} \frac{\ln u}{u^{\lambda} - 1} u^{\lambda_{2} - 1} \,du = O\biggl(\frac{1}{\ln^{\lambda_{2}/2}(m - \xi )}\biggr) \in (0,1), \\ &\stackrel{\smile}{\theta}_{1}(\lambda_{2},m): = \biggl[ \frac{\lambda}{\pi} \sin \biggl(\frac{\pi \lambda_{1}}{\lambda} \biggr)\biggr]^{2} \int_{0}^{\frac{\ln (2 + \eta )}{\ln (m + \xi )}} \frac{\ln u}{u^{\lambda} - 1} u^{\lambda_{2} - 1} \,du = O\biggl(\frac{1}{\ln^{\lambda_{2}/2}(m + \xi )}\biggr) \in (0,1), \end{aligned}$$

(20) reduces to

31 $$\begin{aligned} &\sum_{n = 2}^{\infty} \sum _{m = 2}^{\infty} \biggl\{ \frac{\ln [\ln (m - \xi )/\ln (n - \eta )]}{\ln^{\lambda} (m - \xi ) - \ln^{\lambda} (n - \eta )} + \frac{\ln [\ln (m - \xi )/\ln (n + \eta )]}{\ln^{\lambda} (m - \xi ) - \ln^{\lambda} (n + \eta )} \\ &\qquad{} + \frac{\ln [\ln (m + \xi )/\ln (n - \eta )]}{\ln^{\lambda} (m + \xi ) - \ln^{\lambda} (n - \eta )} + \frac{\ln [\ln (m + \xi )/\ln (n + \eta )]}{\ln^{\lambda} (m + \xi ) - \ln^{\lambda} (n + \eta )} \biggr\} a_{m}b_{n} \\ &\quad> \frac{2\pi^{2}}{[\lambda \sin (\frac{\pi \lambda_{1}}{\lambda} )]^{2}} \Biggl\{ \sum_{m = 2}^{\infty} \biggl[ \bigl(1 - \stackrel{\frown}{\theta}_{1}( \lambda_{2},m)\bigr)\frac{\ln^{p(1 - \lambda_{1}) - 1}(m - \xi )}{(m - \xi )^{1 - p}} \\ &\qquad{}+ \bigl(1 - \stackrel{\smile}{ \theta}_{1}(\lambda_{2},m)\bigr)\frac{\ln^{p(1 - \lambda_{1}) - 1}(m + \xi )}{(m + \xi )^{1 - p}} \biggr]a_{m}^{p} \Biggr\} ^{\frac{1}{p}} \\ &\qquad{}\times \Biggl\{ \sum_{n = 2}^{\infty} \biggl[ \frac{\ln^{q(1 - \lambda_{2}) - 1}(n - \eta )}{(n - \eta )^{1 - q}} + \frac{\ln^{q(1 - \lambda_{2}) - 1}(n + \eta )}{(n + \eta )^{1 - q}} \biggr]b_{n}^{q} \Biggr\} ^{\frac{1}{q}}. \end{aligned}$$

In particular, for $\xi = \eta = 0,\lambda = 1,\lambda_{1} = \lambda_{2} = \frac{1}{2}$, setting

$$\stackrel{\frown}{\theta}_{1}(m): = \frac{1}{\pi^{2}} \int_{0}^{\frac{\ln 2}{\ln m}} \frac{\ln u}{u - 1} u^{ - \frac{1}{2}} \,du = O\biggl(\frac{1}{\ln^{1/4}m}\biggr) \in (0,1), $$

we have the following simple reverse Mulholland-type inequality:

32 $$\begin{aligned} &\sum_{n = 2}^{\infty} \sum _{m = 2}^{\infty} \frac{\ln (\ln m/\ln n)}{\ln (m/n)} a_{m}b_{n} \\ &\quad > \pi^{2} \Biggl[ \sum_{m = 2}^{\infty} \bigl(1 - \stackrel{\frown}{\theta}_{1}(m)\bigr) \frac{\ln^{\frac{p}{2} - 1}m}{m^{1 - p}}a_{m}^{p} \Biggr]^{\frac{1}{p}} \Biggl( \sum_{n = 2}^{\infty} \frac{\ln^{\frac{q}{2} - 1}n}{n^{1 - q}}b_{n}^{q} \Biggr)^{\frac{1}{q}}. \end{aligned}$$

## Conclusions

In this paper, we obtain a new reverse Mulholland’s inequality in the whole plane with a best possible constant factor in Theorems 1–2. Equivalent forms and a few particular cases are considered. The method of real analysis is very important and is the key to prove the reverse equivalent inequalities with the best possible constant factor. The lemmas and theorems can provide an extensive account of this type inequalities.
